# Genetic diversity of Moroccan date palm revealed by microsatellite markers

**DOI:** 10.1590/1678-4685-GMB-2022-0021

**Published:** 2023-06-05

**Authors:** Siham Khoulassa, Haris Khurshid, Mohamed Fokar, Hicham Eliddrissy, Youssef Outeha, Jamal Elfadil, Adil Essarioui, Mohamed Benlyas, Rachid Mentag, Benaissa Elmoualij

**Affiliations:** 1 National Institute of Agronomic Research (INRA), Errachidia, Morocco.; 2 Moulay Ismail University (FSTE/UMI), Errachidia, Morocco.; 3 National Agricultural Research Centre (NARC), Crop Sciences Institute, Islamabad, Pakistan.; 4 Texas Tech University, Center for Biotechnology & Genomics, Lubbock-Texas, USA.; 5 National Institute of Agronomic Research (INRA), Rabat, Morocco.

**Keywords:** Date Palm (*Phoenix dactylifera* L.), phylogeny, genetic diversity, SSR, molecular markers

## Abstract

The genetic diversity between 23 Moroccan date palm cultivars collected from the National Palm Collection at the INRA (National Agricultural Research Institute) experimental field in Zagora was assessed using SSR markers that are specifically designed for date palm. Among the 16 tested SSR, 13 were successfully amplified, and were selected to carry out this study. 208 bands were amplified, ranging from 10 to 25 bands per cultivar with an average of 16 alleles per cultivar. The value of heterozygosity of the studied markers ranged from 0.11 to 0.30. The pairwise genetic distances between those cultivars ranged from 0.06 to 0.46. The hierarchical cluster analysis distributed the 23 genotypes into four different groups of one to ten cultivars.

## Introduction

Knowledge of the genetic diversity of date palms is constantly increasing due to the strategic importance of this tree and the priority of its genetic study. Indeed, the date palm (*Phoenix dactylifera*), monocotyledonous plants of the *Arecaceae* family, is cultivated for different reason: its high productivity, its fruits high nutritional value, its preservation of ecosystems threatened by desertification and its ability to create a microclimate suitable for agriculture in arid conditions. Besides, the total number of date palm trees worldwide is over 150 million trees, spread across 3000 varieties (Sedra, 2013), and distributed in more than 30 different countries (Al-Khayri et al., 2015). 

Regarding genetic diversity, the dioecious character of the date palm and its high number of palm trees result in such variability that it seems impossible to obtain two identical plants by sowing (Brochard, 1974). This variability allowed the selection of many clones, with sometimes very different morphological and physiological characteristics. Due to this extensive genetic heterozygosity of date palms, vegetative propagation is the most used reproduction mode. This technique causes genetic erosion because varieties that appear to be of poor fruit quality, but carry interesting genes, are abandoned. Therefore, it is becoming important to study genetic variability both within and between date palm cultivars, and to use these studies in future date palm conservation and breeding programs.

In Morocco, the date palm is grown mainly in areas on the southern flank of the Atlas Mountains, along rivers and around water points. The climate in these areas is known to be arid pre-Saharian. The phoenicultural national heritage is made up of 6.9 million plants. This heritage is quite diverse, and it is made up of almost 453 genotypes representing 52.3% the other 47.7% is constituted of of seed-derived plants. However, this diversity is increasingly threatened by the climatic conditions which continue to change as well as the traditional agricultural practices used in the oases. In fact, among all the existing genotypes, 12 cultivars are the most appreciated by farmers, namely: Majhoul, Boufeggous, Jihel, Bouskri, Bousthammi Noir, Bouslikhene, Outokdime, Bouittob, Ahardane, Aguelid, Taabdount and Aziza Bouzid (Sedra, 2015). In addition, we find that varieties such as Majhoul and boufeggous, constitute more than 70% of newly cultivated trees, especially in the areas newly cultivated with date palms, created as part of the agricultural strategy adopted by Morocco (Plan Maroc Vert). This kind of “monoculture” (not the literal meaning of single species or varieties, but of a group of varieties with the same genetic characteristics: good date quality and high sensitivity to biotic and abiotic factors) represents a danger for the existence of the species, especially that the other genotypes are only present in wild and abandoned clumps in the outskirts of the palm grove.

Conservation of diversity is essential for phoeniciculture sustainability. To do this, we must first assess its diversity and identify diverse genotypes to be integrated into conservation programs in order to create a reserve or gene pool. This gene pool can be useful in case of the emergence of new threats to this crop such as white scale (*Parlatoria blanchardii*) pest that is threatening date palm plantation in Pakistan (Abbas *et al*., 2014) or nitidulid beetles, *Haptoncus luteolus*, *Carpophilus dimidiatus, C*. *hemipterus, Phyllophaga* spp, red mites (*Tetranychus* spp) reported in date palm plantation in Mexico (Ortiz-Uribe et al., 2019). Above all, it is necessary to assess the genetic relationships between the Moroccan date palm cultivars. One of the preferred molecular markers for the study of genetic diversity and population structure of plants is microsatellites SSR because they are locus-specific, have a high degree of polymorphism, have co-dominant inheritance, and are highly reproducible (Banhos et al., 2008; Chan et al., 2008).

In this study, we assessed genetic diversity among 23 of the most known Moroccan date palm cultivars using SSR markers.

## Material and Methods

### Plant material

The plant material for this study consisted of 23 Moroccan date palm cultivars mainly collected from the National Institute of Agricultural Research Collection in the experimental field in Zagora at the Moroccan eastern south. These cultivars were originally collected from different Moroccan oases (Tafilalet, Figuig, Tata, and Zagora); morphological characteristics of the tree and fruit of each cultivar are described in Table 1.The leaf sampling was conducted randomly on one tree per cultivar. Leaf samples were cleaned to remove dust and microorganisms, and then freeze-dried. 60 mg of dried tissue was ground into a fine powder.

**Table 1 -:** Characteristics of studied cultivars: morphology of the tree and fruit, ripeness, response to Bayoud disease and economic appreciation of the fruit.

	Cultivars	Ripeness of dates	Behavior to Bayoud	Tree vigor	Palm length	Pollination period	Tree productivity	Shape of dates	Date color	Consistence of the date	% of fruit pulp	Commercial appreciation
1	***Sair***	Moderatety late	Resistant	Hight	Medium	End of march	Hight	Cylindrical	Light brown	Semi-dry	Hight	Medium
2	***Tademant***	Seasonal	Resistant	Hight	Very long	End of march	Medium	Cylindrical	Black	Semi-dry	Hight	Medium
3	***Iklane***	Late	Resistant	Hight	Medium	End of march	Hight	Cylindrical	Black	Semi-soft	Very hight	Bad
4	***Bousthammi-Noire***	Moderately late	Resistant	Hight	Very long	End of march	Hight	Opposite ovoid	Black	Soft	Hight	Bad
5	***Bousthammi-Blanche***	Seasonal	Resistant	Hight	Medium	End of march	Medium	Ovoid	Dark brown	Soft	Medium	Bad
6	***Boufeggous-Oumoussa***	Moderately early	Resistant	Very hight	Short	End of march	Hight	Elongated ovoid	Black	Soft	Hight	Bad
7	***Bouzeggar***	Late	Moderately resistant	Hight	Very long	End of march	Hight	Cylindrical	Black	Semi-soft	Hight	Bad
8	***Azegzao***	Moderatety late	Moderately resistant	Low	Short	End of march	Low	Opposite ovoid	Light brown	Semi-dry	Low	Bad
9	***Outoukdim***	Moderatety late	Moderately resistant	Low	Very short	Beginning of April	Medium	Elongated ovoid	Light brown	Dry	Very hight	Medium
10	***Aguelid***	Early	Moderately resistant	Hight	Medium	Beginning of march	Medium	Cylindrical	Light brown	Semi-soft	Medium	Pretty good
11	***Najda***	Seasonal	Resistant	***	Long	Mach to April	***	Elongated ovoid	Light brown	Semi-soft	Very hight	Good
12	***Mah-elbaid***	Moderatety late	Susceptible	Hight	Medium	End of march	Medium	Elongated ovoid	Light brown	Soft	Hight	Pretty good
13	***Mekt***	Seasonal	Susceptible	Medium	Short	End of march	Medium	Elongated ovoid	Black	Soft	Very hight	Bad
14	***Belhazit***	Seasonal	Susceptible	Low	Very short	Beginning of April	Medium	Ovoid	Light brown	Semi-soft	Hight	Medium
15	***Race-Lahmar***	Seasonal	Susceptible	Hight	Medium	End of march	Medium	Ovoid	Light brown	Dry	Medium	Medium
16	***Howa***	Seasonal	Susceptible	Medium	Short	End of march	Medium	Elongated ovoid	Light brown	Semi-dry	Hight	Medium
17	***Boucerdoune***	De saison	Susceptible	Medium	Very short	End of march	Medium	Round	Light brown	Dry	Hight	Medium
18	***Abouijjou***	Seasonal	Susceptible	Medium	Short	End of march	Medium	Cylindrical	Light brown	Dry	Very hight	Medium
19	***Bouittoub***	Moderately late	Susceptible	Low	Short	End of march	Medium	Opposite ovoid	Light brown	Dry	Hight	Medium
20	***Bousekri***	Moderately late	Susceptible	Low	Short	End of march	Medium	Elongated ovoid	Greenish brown	Dry	Medium	Medium
21	***Oum-nhal***	Moderately late	Susceptible	Hight	Medium	End of march	Medium	Elongated ovoid	Light brown	Semi-soft	Low	Pretty good
22	***Boufegous***	Seasonal	Very susceptible	Medium	Very short	End of march	Hight	Ovoid	Dark brown	Soft	Very hight	Good
23	***Majhoul***	Late, moderately late, seasonal	Very susceptible	Medium	Short	End of march	Very hight	Elongated ovoid	Dark brown	Semi-soft	Very hight	Very good

### DNA extraction

Extraction of genomic DNA was done using the CTAB method (Richards et al., 1997) with some modifications, then Qubit 3 fluorometer was used for quantification. A 0,8% agarose gel electrophoresis was used to assess DNA quality, migration was for 20 minutes at 120 V. 

### SSR amplification

Sixteen primer pairs of date palm specific SSR (Billotte et al., 2004) were tested (Table 1). PCR reactions were performed in 20 µl reaction mixture containing: 50 ng of DNA, 0.2 mM of DNTP mix (Thermo scientific), 0.25 mM MgCl2 (Thermo Scientific), 0.5 U of DreamTaq DNA polymerase (Thermo Scientific) and 0.2 mM of primers. Amplifications were performed in a Thecne TC-4000 thermal cycler using the following program: a 2 min denaturation step at 95 °C followed by 35 cycles of 30 s at 95 °C, 30 s at 52-60 °C (according to the primer pair Tm), 60 s at 72 °C, and 7 min at 72 °C for the final extension. PCR products were separated on 6% acrylamide gel, stained with ethidium bromide, then visualized and photographed using a trans-illuminator and Clara vision gel documentation system.

### Data analysis

The band’s molecular weight was measured using Gel Analyzer software and scored for each cultivar as a 0 or 1 (absent or present) in excel sheet. Marker diversity indices such as Effective alleles, Heterozygosity, and Shannon’s index were computed for all the SSR markers to examine their usefulness in dissecting genetic variation in studied genotypes. STRUCTURE 2.3.4 program was used to determine existing population structure and classify our 23 date palm genotypes into an optimum number of populations on the basis of SSR markers data, and estimate population allele frequencies (Pritchard et al., 2000). The results were then submitted to the online module of the STRUCTURE HARVESTER (Earl et al., 2012). This was also visualized using biplot analysis in software showing the distribution of 23 genotypes into populations represented by colored bars. 

The SSR data of genotypes along with respective population names (pop1, pop2, pop3, pop4) as found by STRUCTURE software was then analyzed in GeneAlex software for analysis of molecular variance (AMOVA). 

In addition to STRUCTURE-based classification, pairwise genetic distances were computed for all the 23 genotypes based on their SSR markers presence-absence data. R language package “Vegan” was used to calculate Jaccard’s similarity indices. The minimum number between two genotypes means these were similar while the maximum number means that they were diverse.

Furthermore, the Jaccard’s similarity index was used to generate a dendrogram using the “Dendextend” package in R language (Galili, 2015). The package performed hierarchical cluster analysis using the “Complete” method and distributed 23 genotypes into different groups.

## Results

Among the 16 studied primers, 13 revealed polymorphic bands and were used to assess genetic relationships within the 23 tested cultivars (Table 2). Marker diversity was computed for all the SSR markers to examine their usefulness in dissecting genetic variation in studied genotypes (Table 2). The total number of bands was 208 loci detected by 13 SSR pairs for the 23 date palm Moroccan cultivars. The allele numbers ranged from 10 bands per primer for the locus mPdCIR017 to 25 for the locus mPdCIR032 with an average of 16 bands per primer. The value of heterozygosity ranged from 0.11 for the locus mPdCIR16 to 0.30 for the locus mPdCIR035. 

**Table 2 -:** Characteristics of the 16 microsatellite loci studied: name, repeat motif, clone size, working temperature, their sequences, number of alleles, number of effective alleles, heterozygosity, and Shannon’s information index.

SSR Locus	Repeat motif	Clone size (bp)	Tm (°C)	Primer sequences (5’-3’)	N° of alleles	Effective alleles	Heterozygosity	Shannon’s index
***mPdCIR010***	*(GA)22*	*180*	*55*.*9*	*F:ACCCCGGACGTGAGGTG R:CGTCGATCTCCTCCTTTGTCTC*	13	1.340	0.218	0.343
***mPdCIR015***	*(GA)15*	*253*	*51*.*6*	*F:AGCTGGCTCCTCCCTTCTTA R:GCTCGGTTGGACTTGTTCT*	10	1.444	0.272	0.425
***mPdCIR016***	*(GA)14*	*209*	*51*.*7*	*F:AGCGGGAAATGAAAAGGTATR:ATGAAAACGTGCCAAATGTC*	19	1.161	0.113	0.191
***mPdCIR025***	*(GA)22*	*269*	*49*.*3*	*F:GCACGAGAAGGCTTATAGTR:CCCCTCATTAGGATTCTAC*	17	1.413	0.266	0.418
***mPdCIR032***	*(GA)19*	*376*	*51*.*5*	*F:CAAATCTTTGCCGTGAGR:GGTGTGGAGTAATCATGTAGTAG*	25	1.246	0.184	0.316
***mPdCIR035***	*(GA)15*	*341*	*53*.*9*	*F:ACAAACGGCGATGGGATTACR:CCGCAGCTCACCTCTTCTAT*	14	1.492	0.304	0.469
***mPdCIR044***	*(GA)19*	*340*	*51*.*7*	*F:ATGCGGACTACACTATTCTAC R:GGTGATTGACTTTCTTTGAG*	13	1.316	0.212	0.349
***mPdCIR048***	*(GA)32*	*439*	*51*.*4*	*F:CGAGACCTACCTTCAACAAAR:CCACCAACCAAATCAAACAC*	14	1.418	0.265	0.419
***mPdCIR050***	*(GA)21*	*568*	*48*.*5*	*F:CTGCCATTTCTTCTGACR:CACCATGCACAAAAATG*	*-*	*-*	*-*	*-*
***mPdCIR057***	*(GA)20*	*360*	*55*.*4*	*F:AAGCAGCAGCCCTTCCGTAGR:GTTCTCACTCGCCCAAAAATAC*	11	1.468	0.286	0.440
***mPdCIR063***	*(GA)17*	*301*	*49*.*8*	*F:CTTTTATGTGGTCTGAGAGAR:TCTCTGATCTTGGGTTCTGT*	16	1.218	0.159	0.270
***mPdCIR070***	*(GA)17*	*265*	*48*.*7*	*F:CAAGACCCAAGGCTAACR:GGAGGTGGCTTTGTAGTAT*	18	1.413	0.273	0.434
***mPdCIR078***	*(GA)13*	*260*	*49*.*6*	*F:TGGATTTCCATTGTGAGR:CCCGAAGAGACGCTATT*	16	1.290	0.210	0.348
***mPdCIR085***	*(GA)29*	*375*	*50*.*4*	*F:GAGAGAGGGTGGTGTTATT R:TTCATCCAGAACCACAGTA*	*-*	*-*	*-*	*-*
***mPdCIR090***	*(GA)26*	*269*	*48*.*6*	*F:GCAGTCAGTCCCTCATAR:TGCTTGTAGCCCTTCAG*	*-*	*-*	*-*	*-*
***mPdCIR093***	*(GA)16*	*230*	*51*.*8*	*F:CCATTTATCATTCCCTCTCTTGR:CTTGGTAGCTGCGTTTCTTG*	21	1.251	0.181	0.304

**Figure 1 -:**
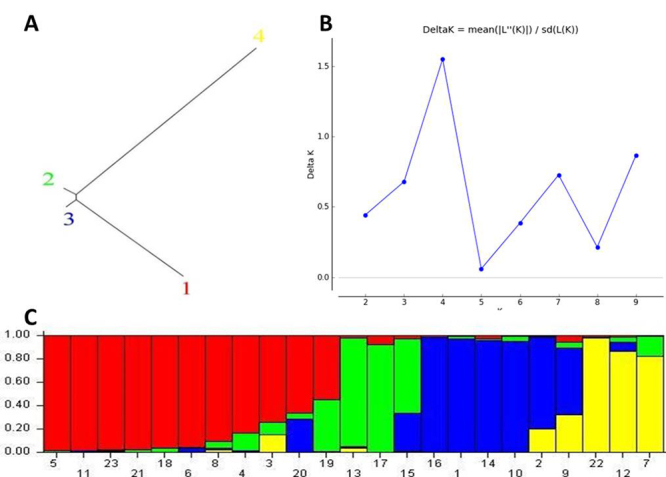
Population structure and membership probability of 23 date palm cultivars based on SSR polymorphic markers. The model-based clustering was constructed using STRUCTURE 2.3.4 software. A. STRUCTURE based neighbour joining tree derived from SSR markers showing the genetic relationships among 23 Moroccan date palm cultivars. B. The highest peak of K = 4 determines the number of populations in our study. C. Population structure distributing 23 date palm cultivars into 4 populations.

The online module of STRUCTURE harvester revealed that optimum number of populations was four or Δk=4 (Highest peak of Figure 1A,B). Hence, the 23 cultivars studied are devided into four groups, the first groupe (Pop1) was made up of ten cultivars: *Iklane, Bousthammi-noire, Bousthammi-blanche, Boufeggous-oumoussa, Azegzao, Najda, Abouijjou, Bousekri, Oum-nhal*, and *Majhoul*. The second groupe (Pop 2) consisted of four cultivars: *Mekt, Race-lahmar, Boucerdoune*, and *Bouittoub*. The third groupe (Pop3) was composed of six cultivars *Sair, Tademant*, *Outoukdim, Aguelid*, *Belhazit*, and *Howa*. The fourth and last groupe (Pop4) was made up of three cultivars *Bouzeggar, Mah-elbaid,* and *Boufegous*. This result was also visualized using biplot analysis in software showing distribution of 23 genotypes into 4 populations represented by colored bars (Figure 1C). 

The SSR data of genotypes along with respective population name (pop1 to 4) as found by STRUCTURE software was then analysed in GeneAlex software for analysis of molecular variance (AMOVA). The p < 0.01 showed that there is significant difference between all the populations. The genetic distance between these populations was shown by distance matrix: Pop 2 and Pop 4 were more diverse from each other with a maximum distance of 0.453. The minimum distance of 0.017 was recorded between Population 3 and population 4.

The pairwise genetic distances were computed for all the 23 genotypes based on their SSR markers presence absence data using R language package to calculate Jaccard’s similarity indexes (Table 3). Indexes are ranging from 0.06 to 0.46 for the studied cultivars. According to these indexes, cultivar *Mah-Elbaid* is genetically close to cultivars *Boufeggous* and *Bouzeggar* with the highest genetic similarity of 0.46 and 0.45 respectively. The most distant cultivars are Howa/ Oumnhal with a similarity index of 0.06 followed by *Majhoul/ Oumnhal* and *Bouzeggar/ Bousthammi* noir with an index of 0.07. 

**Table 3 -:** Jaccard similarity indices between the 23 studied Moroccan cultivars.

		1	2	3	4	5	6	7	8	9	10	11	12	13	14	15	16	17	18	19	20	21	22	23
***Sair***	1	1																						
***Tademant***	2	0.34	1																					
***Iklane***	3	0.2	0.23	1																				
***Bousthammi-noire***	4	0.08	0.12	0.09	1																			
***Bousthammi-blanche***	5	0.31	0.26	0.21	0.14	1																		
***Boufeggous-oumoussa***	6	0.21	0.27	0.19	0.27	0.3	1																	
***Bouzeggar***	7	0.28	0.31	0.24	0.07	0.24	0.2	1																
***Azegzao***	8	0.19	0.17	0.17	0.11	0.17	0.09	0.11	1															
***Outoukdim***	9	0.3	0.25	0.14	0.1	0.19	0.19	0.27	0.14	1														
***Aguelid***	10	0.39	0.4	0.21	0.12	0.26	0.22	0.29	0.16	0.26	1													
***Najda***	11	0.26	0.17	0.22	0.19	0.31	0.24	0.14	0.11	0.13	0.18	1												
***Mah-elbaid***	12	0.34	0.39	0.26	0.13	0.25	0.25	0.45	0.15	0.33	0.35	0.17	1											
***Mekt***	13	0.36	0.28	0.28	0.12	0.26	0.18	0.3	0.19	0.29	0.36	0.2	0.33	1										
***Belhazit***	14	0.36	0.3	0.15	0.13	0.24	0.23	0.2	0.19	0.18	0.33	0.19	0.3	0.26	1									
***Race-lahmar***	15	0.34	0.27	0.23	0.16	0.26	0.25	0.23	0.12	0.23	0.31	0.17	0.28	0.28	0.28	1								
***Howa***	16	0.31	0.3	0.24	0.11	0.21	0.14	0.18	0.26	0.17	0.27	0.16	0.21	0.32	0.24	0.28	1							
***Boucerdoune***	17	0.31	0.34	0.19	0.16	0.25	0.18	0.29	0.16	0.24	0.3	0.25	0.32	0.29	0.23	0.23	0.28	1						
***Abouijjou***	18	0.24	0.21	0.26	0.22	0.25	0.2	0.14	0.15	0.16	0.21	0.32	0.21	0.26	0.22	0.24	0.28	0.27	1					
***Bouittoub***	19	0.23	0.24	0.2	0.12	0.26	0.18	0.16	0.16	0.18	0.21	0.21	0.2	0.27	0.24	0.18	0.26	0.34	0.31	1				
***Bousekri***	20	0.28	0.24	0.18	0.16	0.3	0.22	0.23	0.19	0.12	0.27	0.3	0.22	0.29	0.21	0.29	0.28	0.33	0.34	0.28	1			
***Oum-nhal***	21	0.09	0.1	0.13	0.11	0.13	0.12	0.14	0.14	0.08	0.11	0.14	0.11	0.1	0.09	0.09	0.06	0.09	0.09	0.08	0.2	1		
***Boufegous***	22	0.28	0.3	0.25	0.15	0.17	0.24	0.4	0.14	0.29	0.23	0.18	0.46	0.26	0.23	0.25	0.17	0.26	0.18	0.21	0.17	0.11	1	
***Majhoul***	23	0.18	0.18	0.19	0.2	0.22	0.33	0.14	0.13	0.14	0.16	0.26	0.16	0.18	0.19	0.22	0.19	0.22	0.27	0.27	0.21	0.07	0.25	1

**Figure 2 -:**
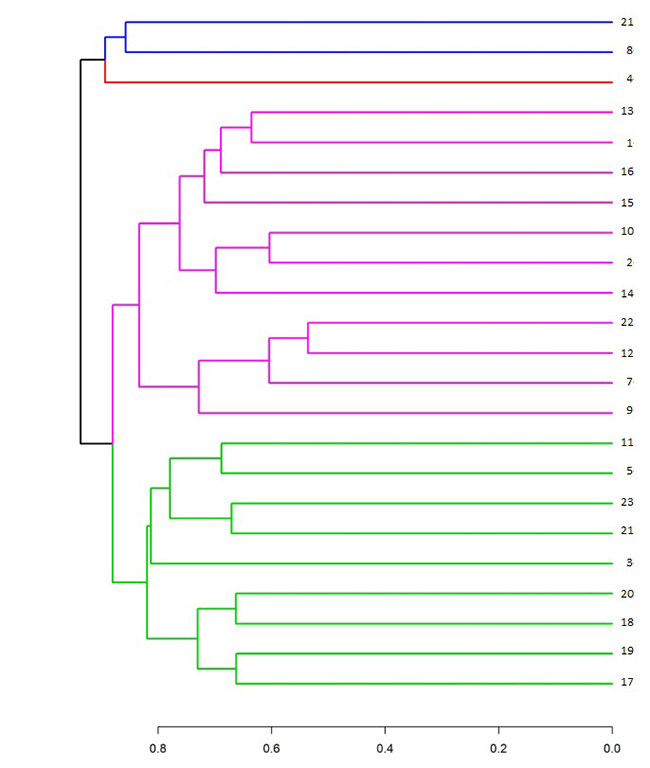
Dendrogram of genetic similarity of the 23 studied cultivars. 1- Sair, 2- Tademant, 3- Iklane, 4- Bousthammi-noire, 5- Bousthammi-blanche, 6- Boufeggous-oumoussa, 7- Bouzeggar, 8- Azegzao, 9- Outoukdim, 10- Aguelid, 11- Najda, 12- Mah-elbaid, 13- Mekt, 14- Belhazit, 15- Race-lahmar, 16- Howa, 17- Boucerdoune, 18- Abouijjou, 19- Bouittoub, 20- Bousekri, 21- Oum-nhal, 22- Boufegous, 23-Majhoul.

The dendrogram (Figure 2) was generated using R language in the basis of the Jaccard similarity indexes. It shows the distribution of the 23 cultivars into four groups represented by different colors. The first group was constituted by cultivars *Oum-nhal* and *Azegzao*; the second group constituted only by cultivar *Bousthammi-noire*; the third group consisted of 11 cultivars: *Mekt, Sair, Howa, Race-lahmar, Boucerdoune, Aguelid, Tademant, Belhazit, Boufegous, Mah-elbaid, Bouzeggar,* and *Outoukdim;* the last group was constructed by nine cultivars: *Iklane, Bousthammi-blanche, Boufeggous-oumoussa, Najda, Abouijjou, Bousekri, Bouittoub*, and *Majhoul*.

## Discussion

Breeding is an important step in the conservation and sustainable development of species. For fruit trees, the breeding is very challenging because of the trees long life cycles and the dioecious characters, as in date palm case; a perennial, dioic, monocotyledon tree; which can live for hundreds of years. In these cases, understanding the genetic relationships between varieties is an important tool in facilitating genetic selection work. This study contributes to this understanding and will be added to the works already carried out to enrich them. 

The SSR primers used in this study to assess genetic diversity of Moroccan date palm cultivars showed good amplification and polymorphism. A total of 208 bands were detected by thirteen pairs of SSR primers for the 23 cultivars studied. The number of alleles per locus varied from 10 for the locus mPdCIR017 to 25 for the locus mPdCIR032. Previous works showed that these primers have an important variation in alleles per locus numbers. According to Bodian et al. (2012), a maximum number of alleles (11) was detected by MdPCIR050; ranging between 4 and 11, whereas Ahmed et al. (2021) reported a number of alleles per locus ranging from 5 for mPdCIR032 to 16 for mPdCIR085 and mPdCIR093. Also, Aljuhani (2016) reported a number of alleles ranging from 6 to 15 per locus and maximum (15) number of alleles amplified by primer MdPCIR015. However, Zehdi et al. (2004) found a number of alleles per locus ranging from 4 to 10 when examining 46 Tunisian date palm accessions using 14 microsatellite loci, whereas Ahmed and Al-Qaradawi, (2009) reported a number of alleles per locus varied from 3 for the locus mPdCIR016 to 6 for the locus mPdCIR032 by examining 15 Qatari date palm cultivars using 16 SSR primers.

Statistical analysis showed that the genetic similarities between cultivars are quite variable (ranging from 0.06 to 0.46). These genetic similarities are lower than those found by Zehdi et al. (2004) (ranging between 0.3008 and 0.7885), by Ahmed and Al-Qaradawi (2009) (ranging from 0.00 to 0.75), by Bodian et al. (2012) (ranging between 0.16 and 0.76) and by Aljuhani (2016) (ranging from 0.25 to 0.95). These values suggest that the Moroccan cultivars chosen for this study are genetically close; mainly because of the interspecific crosses which represented the common ways of multiplication in Moroccan oases, in traditional palm groves, and the fact that the cultivars in INRA’s experimental field were selected for their morphological characteristics, particularly their fruit quality. Whereas some of them are closer than the other, ‘Mah-Elbaid’ is genetically the closest to ‘Boufeggous’ with the highest similarity value of 0.46. While ‘Howa’ and ‘Oum-nhal’ are the most distant, with a similarity value of 0.06. 

The relationships between the studied Moroccan date palm cultivars are shown in the dendrogram (Figure 2). The dendrogram generated four major clusters. In cluster one, Azegzao is clustered together with Oum-nhal. Bousthammi-noire was classified distinctively from all cultivars. Other cultivated varieties (Sair, Tademant, Bouzeggar, Outoukdim, Aguelid, Mah-elbaid, Mekt, Belhazit, Race-lahmar, Howa, and Boufegous) were found in cluster three. Iklane, Bousthammi-blanche, Najda, Boucerdoune, Abouijjou, Bouittoub, Bousekri and Majhoul were clustered in a distinct subset. Common cultivars with Bodian et al. (2012; 2014) studies showed different distribution, probably due to the difference of origins. Confronting this distribution to morphological characteristics such as fruits shapes and colors, or cultivars behavior to Bayoud disease … shows no significant relation to any of these characters. 

These results will be useful for our work on the study of date palm genetic diversity in the Moroccan oasis. We will be able to use them as a standard to compare randomly sampled date palm trees throughout the Tafilalet Valley. 
